# The Medical Society of the County of Erie

**Published:** 1893-02

**Authors:** Eli H. Long

**Affiliations:** Secretary


### THE MEDICAL SOCIETY OF THE COUNTY OF ERIE.

*Annual Meeting, January 10, 1893.*

Reported by ELI H. LONG, M. D., Secretary.

MORNING SESSION.

The meeting was called to order in the Y. M. C. A. Building, at 10.30 A. M. The President, Dr. William Warren Potter, in the chair.

The minutes of the previous meeting were read and approved.

The Committee on Membership reported favorably upon the following-named candidates, who were duly elected members of the society, namely: Eleanor McAllister, Loren H. Staples, Carlos E. Bowman, Mary T. Greene, H. C. Leonhardt, Jane W. Carroll, Robert S. Hambleton, Frank J. Thornbury, Henry F. Carter, and

Alfred E. Diehl. Those present were formally introduced to the society.

The Committee on Membership further reported the receipt of applications for membership from the following-named physicians: Francis T. Metcalfe, Franklin C. Gram, Clarence A. Tyler, James Stoddart, F. G. Moehlan, J. F. Whitwell, G. W. Wende, Duncan Sinclair, Geo. J. Hearne, Edward R. Wiser, Albert F. Erb, Harry Corwin Jones, Cyrus S. Siegfried, Chas. E. Long, Geo. A. Himmelsbach, Edward L. Frost, and E. J. Meyer. These applications were directed to lie over until the June meeting, under the rules.

It was moved by Dr. John Cronyn, and seconded, that the order of business be suspended so as to allow the President's address to be presented just after completion of the routine business of the society. Carried unanimously.

Dr. W. C. Callanan, of the Committee on Metric System, reported that little had been done by the committee since the last meeting, but urged a more extensive adoption of the system by members of the society.

The committee was continued by unanimous consent.

The Secretary moved that the new Committee on Programme be instructed to arrange as one part of the programme for the next meeting a discussion upon the Use of the Metric System. Carried.

The Treasurer presented his report, which showed a balance in the treasury of $256.99. The report was referred to an Auditing Committee, consisting of Drs. O'Brien and Callanan, and upon their favorable report as to its correctness, the report was adopted.

The Librarian presented his report as follows:

LIBRARIAN'S REPORT.

January 10, 1893.

*To the Medical Society of the County of Erie:*

On account of peculiar circumstances, your library is not meeting nor fulfilling the expectations of its usefulness.

During the year just closed, not a single application has been made to your librarian for information regarding, nor application for, books in it.

Had application been made for information, I could have given but little. I have searched for a catalogue, but have found none. On account of the limited shelf-room, the books are placed in positions most convenient for storing, and which are incompatible with convenience of inspection or access. In this chaotic condition I have not been able to catalogue and arrange the books.

Your library should be in such location and condition that its contents may be available at any time. It goes without saying, that a practitioner having a case on which he wants information cannot spend time in hunting up the librarian or going beyond the center of the city.

I would recommend that this society instruct the librarian to find locations for its library and enter into negotiations regarding each, such negotiations to pend during report to and action by the society at its earliest possible moment.

Respectfully submitted.

CHARLES A. RING, *Librarian.*

The report was ordered received and placed on file.

Dr. M. D. Mann, in discussion of the report, suggested that the library be entrusted to the keeping of the University of Buffalo, with the understanding that the institution named keep up the files of periodicals now taken by the society.

Dr. H. R. Hopkins moved, that in view of this offer by the University of Buffalo, the whole matter be referred to the new President and Librarian, with power to act.

Carried.

The Board of Censors being without a chairman, owing to the resignation of Dr. Storck, no regular report was presented. Dr. Walsh, of the board, and Dr. Krug, who had been appointed acting-chairman for a special purpose, made verbal statements concerning a case of irregular practice which was now before the grand jury.

The Secretary presented the resignation of Dr. B. H. Grove as a delegate to the State Medical Society, as he has been elected a permanent member thereof, which was accepted.

A motion having been made for the appointment of a Nominating Committee, the President stated that if there was no objection he would appoint a Nominating Committee of five members to nominate all officers, except President and Vice-President. Drs. O'Brien, Rochester, Coakley, Lapp, and MacPherson, were appointed as such committee.

The society then proceeded to the election of President and Vice-President. The Chair appointed as tellers, Drs. Walsh and Benedict. For the office of President, Dr. Cronyn nominated Dr. Wm. H. Gail, and Dr. Walsh nominated Dr. Jno. Parmenter. The ballot was then taken with the following result: Of the fifty-five votes cast, Dr. Parmenter received twenty-nine, Dr. Gail received twenty-five, and Dr. Mann received one. Dr. Parmenter was declared elected.

For the office of Vice-President were nominated, Dr. Chas. G. Stockton, Dr. A. H. Briggs, Dr. Herman Mynter, Dr. Wm. H. Jackson, Dr. Jno. A. Pettit. Drs. Stockton and Pettit declined. A ballot was then taken with the following result: Of the fifty-seven votes cast, Dr. Jackson received twenty-three, Dr. Mynter received nineteen, Dr. Briggs received fifteen. No candidate having received a majority, the CHAIR ordered a second ballot, which resulted as follows: Of the fifty-seven votes cast, Dr. Jackson received thirty-two, Dr. Mynter received seventeen, Dr. Briggs eight. Dr. Jackson was declared elected.

Next in order, according to previous decision of the society, was the delivery of the Annual Address by the PRESIDENT, during which Dr. Cronyn was invited to occupy the chair.

After the address, Dr. STOCKTON moved the adoption of the following:

*Resolved,* That the thanks of the society are due and are hereby tendered to the retiring President, not only for the excellent address presented, but also for the efficient manner in which he has conducted the affairs of the society during his term of office.

Dr. CALLANAN moved to amend, that a copy of the address be requested for publication.[1] Amendment accepted and resolution unanimously adopted.

Dr. HOPKINS then presented and moved the adoption of the following:

*Resolved,* That the thanks of this society are due, and are hereby tendered, our distinguished member, Hon. Jacob Goldberg, M. D., member of the Assembly, for his efforts in preserving from pernicious amendment the wise and beneficent medical law of this State.

*Resolved,* That we also gladly recognize the valuable services, in the same most worthy cause, of the Hon. R. P. Bush, M. D., member and Speaker of the Assembly.

*Resolved,* That we call upon our members of Senate and Assembly to use all honorable means to guard and preserve our medical laws, as the promoters and protectors of the public health.

*Resolved,* That a suitable letter be sent to each county medical society, requesting them to urge the Medical Society of the State of

1. The President's address, under this order, is printed in full. See page 401, this journal.

New York to memorialize the Legislature to the end that, hereafter, in their own interests and in the interests of public health, no amendment to our medical law be received until the same shall have been considered by the Regents of the University and by the medical profession as represented in our incorporated State and local medical societies.

Carried.

The society then took a recess until 3.15 P. M.

AFTERNOON SESSION.

The afternoon session was called to order, at 3.15 P. M., by the President.

Dr. IRVING M. SNOW presented to the society a case of congenital heart disease.

Under Miscellaneous Business, Dr. C. A. RING presented and moved the adoption of the following:

WHEREAS, The office of Censor of a county medical society is of the highest importance to the public, affording it protection against the practice of medicine by incompetent or unscrupulous persons ; and

WHEREAS, The office is interested in the highest medical education and proficiency ; and

WHEREAS, The faithful performance of the duties of the office require that much time be given, for which there is no financial return; and

WHEREAS, In the exercise of the duties of the office, enmities form from which may come personal discomfort ; and

WHEREAS, Perfect moral courage, integrity, and rectitude are required in action which may have for its consideration either a member of this society or some one at variance with the body-politic ; and

WHEREAS, For the last twelve years, as member of the Board of Censors of this society, and as Chairman thereof, Doctor Edward Storck has filled the requirements of duties as member of that Board, and as its chief executive officer ; therefore,

*Resolved*, That the Medical Society of the County of Erie hereby expresses its entire approval of the manner in which Doctor Edward Storck has discharged the duties of member of the Board of Censors, and as Chairman thereof.

That this society tenders Doctor Edward Storck a vote of thanks for the efficient manner in which he has served this society.

That this society learns with regret of his determination not to accept office on the Board of Censors.

That a copy of these resolutions, under the seal of this society, and of the signatures of its proper officers, be sent to Doctor Edward Storck.

That a copy of these resolutions be furnished the daily press and the publication thereof be requested.

Adopted unanimously.

Dr. Ring also offered the following :

Whereas, The institution and maintenance of quarantine is for the protection of the country from a common danger ; and

Whereas, All the people of the country, those of the interior as well as those on the borders or frontiers, are equally interested in the results of its efficiency, and should bear a proportionate share of its maintenance ; therefore,

*Resolved,* That the Medical Society of the County of Erie is in favor of a system of quarantine to be instituted and maintained by the Federal Government.

*Resolved,* That the delegates of this society to the Medical Society of the State of New York be instructed to bring the subject-matter of these resolutions before the Medical Society of the State.

*Resolved,* That to the members of Congress from this county a copy of these resolutions be sent, and that they be requested to use all honorable means to carry into effect such measures as may be in accord herewith.

Carried.

The Nominating Committee presented the following report: For Secretary, Dr. Eli H. Long ; for Assistant Secretary, Dr. Geo. F. Cott; for Treasurer, Dr. Edward Clark; for Assistant Treasurer, Dr. Eugene A. Smith ; for Librarian, Dr. Chas. A. Ring ; for Committee on Membership, Dr. John A. Pettit, Dr. Jas. W. Putnam, Dr. Geo. W. MacPherson ; for Censors, Dr. P. W. Van Peyma, Dr. Henry Lapp, Dr. J. F. Krug, Dr. J. H. Potter, Dr. A. L. Benedict; for Delegates to the Medical Society of the State of New York, Dr. Wm. H. Bergtold, Dr. Jno. J. Walsh, Dr. Wm. C. Callanan.

Upon motion by Dr. Hopkins, the report was adopted and the nominees declared elected.

Dr. Hopkins presented and moved the adoption of the following :

*Resolved,* That it is the sense of this society that the practice known as that of the Keely Gold Cure is not such as to claim the support of this society ; and that we do not look with favor upon the connection with these so-called institutes of members of this society.

Dr. Benedict moved to amend the resolution by adding the following :

*Resolved,* That this society expresses its disapprobation of admitting to membership or continuing in membership, in County Medical Societies, persons connected with medical institutions which advertise in the daily press or in other ways than in journals intended to circulate among medical men.

Amendment adopted.

Original resolution adopted as amended.

The scientific programme was then taken up.

Papers upon Hydatid Disease were read by Dr. Roswell Park, Dr. H. U. Williams, and Dr. Herman Mynter.

The following members led a discussion upon the Relation of Gonorrhea to Pelvic Diseases in Women: Dr. M. D. Mann, Dr. M. A. Crockett, Dr. C. C. Frederick. The discussion was also participated in by Drs. Mynter, Rochester, Hayd, the President, and Pryor, and closed by Drs. Mann, Crockett, and Frederick.

Dr. R. S. Myers presented the following obituary notice of Dr. Lewis C. Carmer:

### MEMOIR OF DR. LEWIS C. CARMER, M. D.

By R. S. MYERS, M. D.

Dr. Lewis C. Carmer was born in the Town of Clarence, Erie County, New York, June 16, 1858, on a farm where his father and mother had settled on at an early day, and where they now reside.

Dr. Carmer's early years were spent working on the farm during the summer season and attending the country school during the winters. When sixteen years of age he was admitted as a student at the State Normal School at Buffalo, N. Y., where he prosecuted his academical and classical studies for four years, after which he remained at home on the farm for one year. He then commenced his medical studies in the Medical Department of the University of Michigan, at Ann Arbor, and, after the attendance of three full courses of lectures, he received his degree of M. D. from that institution in 1883.

Thus equipped by thorough mental and professional culture, he at once engaged in the practice at Williamsville, this county.

He was married to Julia Idsardi, June 16, 1886, who survives him. He continued to practice at Williamsville up to a short time of his death, which occurred at the home of his wife's father, T. E. Idsardi, in the Town of Lancaster, N. Y., July 11, 1892. He died from acute phthisis, in the thirty-fifth year of his age.

Dr. Carmer was a highly educated gentleman, a conscientious and judicious practitioner, and honorable and exemplary in all his social relations. He had a large country practice, and had greatly endeared himself to his patients by his kindness, attention, and professional skill.

He was a member of the Erie County Medical Society, and also a zealous member of the Gross Medical Club, of which he served as President. His future was full of promise, and by his death the profession and science, equally with family and friends, have sustained an irreparable loss.

The CHAIR appointed, as a Committee on Programme, Drs. J. H. Pryor, DeLancey Rochester, and Wm. C. Krauss, with the President and Secretary *ex-officisis.*

Upon motion by Dr. HAYD, the Secretary and Treasurer were each allowed the sum of $50 for services during the past year.

Adjourned, at 6.15 P. M., without day.

